# Capacity-building strategy for next-generation mental health research: embedding a national network infrastructure to grow mental health researcher capabilities and mental health lived-experience research leaders

**DOI:** 10.1136/bmjment-2025-301554

**Published:** 2025-03-25

**Authors:** Dana Jazayeri, Michelle Banfield, Caley Tapp, Caroline Tjung, Tegan Stettaford, Victoria Stewart, Giulietta Valuri, Terence Chong, Patricia Cullen, Martina McGrath, Rebecca Cooper, Amanda J Wheeler, Amanda L Neil, Steve Kisely, Jill Bennett, David Preen, Sandra Eades (AO), Lena Sanci, Emma Baker, Victoria J Palmer

**Affiliations:** 1 The ALIVE National Centre for Mental Health Research Translation, The University of Melbourne, Melbourne, Victoria, Australia; 2 Centre for Mental Health Research, National Centre for Epidemiology and Public Health, College of Health and Medicine, Australian National University, Canberra, Australian Capital Territory, Australia; 3 Faculty of Medicine, School of Public Health, The University of Queensland, Herston, Queensland, Australia; 4 School of Medicine and Public Health, College of Health, Medicine and Wellbeing, The University of Newcastle, Callaghan, New South Wales, Australia; 5 School of Pharmacy and Medical Sciences, Griffith University, Southport, Queensland, Australia; 6 School of Population and Global Health, The University of Western Australia, Perth, Western Australia, Australia; 7 Faculty of Medicine, Deparment of Psychiatry, Melbourne Medical School, Dentistry and Health Science, The University of Melbourne, Melbourne, Victoria, Australia; 8 Department of Psychiatry, St Vincent’s Hospital Melbourne, Fitzroy, Victoria, Australia; 9 Faculty of Medicine and Health, School of Population Health, University of New South Wales, Sydney, New South Wales, Australia; 10 Faculty of Medicine, Dentistry and Health Sciences, The University of Melbourne, Melbourne, Victoria, Australia; 11 Faculty of Medicine, Deparment of Psychiatry, Melbourne Medical School, Dentistry and Health Sciences, The University of Melbourne, Melbourne, Victoria, Australia; 12 Department of Psychiatry and Behavioral Sciences, Boston Children’s Hospital, Harvard Medical School, Boston, Massachusetts, USA; 13 Menzies Institute for Medical Research, University of Tasmania, Hobart, Tasmania, Australia; 14 Faculty of Arts, Big Anxiety Research Centre, Design and Architecture, University of New South Wales, Paddington, New South Wales, Australia; 15 Faculty of Medicine, Primary Care Mental Health Research Program, The Department of General Practice and Primary Care, Dentistry and Health Sciences, The University of Melbourne, Melbourne, Victoria, Australia; 16 Faculty of Arts, Australian Centre for Housing Research, School of Social Sciences, Law and Economics, The University of Adelaide, Adelaide, South Australia, Australia

## Abstract

Internationally, capacity building for mental health implementation and translation research has lagged. A review of literature found initiatives since 2008 indicating limited dedicated attention to growing capabilities of early-to-mid-career mental health researchers, and little reporting of tailored career pathways and skills growth. Significant gaps in capacity building thus exist. This perspective article describes a networked infrastructure for a capacity building strategy of the Australian-based ALIVE National Centre for Mental Health Research Translation. The Centre was funded as a special initiative in mental health with an initial five-year investment. In 2022, the Centre established the first national, cross-disciplinary mental health Next Generation Researcher Network, including a tailored Lived-Experience Research Collective with the aim to grow future research leaders and establish career pathways embedded within the research activities of the Centre. After three years of operation, membership is upward of 280 people in the Next Generation Researcher Network and more than 250 people for the Collective. Specific components implemented as part of the strategy include a central coordination hub, coleadership approaches, coresearch models, tailored traineeships, skills-building through short courses and learning events, cocreation of resources, an online peer discussion platform and annual seed funding schemes. A continuous capacity-building strategy is critical for advancing global research agendas to improve mental health implementation and translation outcomes. Success requires network infrastructure to ensure research methodologies advance, and research addresses the priorities of people most impacted, and early and mid-career researcher capabilities across all research settings connected with universities and service sectors grow.

## Introduction

 Coordinated efforts to grow the capacity of early and mid-career university-based researchers are ad hoc, and where they have been offered, they are usually time limited. A preliminary review of the literature confirmed this, with eleven capacity building initiatives in mental health research reported for early and mid-career researchers since 2008.^1^,^11^ Of the eleven, five initiatives were in low-income and middle-income countries and typically aimed to fill service gaps and thus focused on clinician researchers to increase system resources. Six initiatives were in high-income countries. Two addressed capacity building in older-age mental health research: (1) was a postdoctoral training programme conducted across multiple research institutions sharing responsibilities;^1^ and, (2) described a mentoring case study with only one participant.^4^ The third paper was a school of psychology conference event^9^ and the fourth a career development programme for psychiatry.^5^ The fifth paper described training 31 early-career to professorial level researchers over a two-year period in the Implementation Research Institute, established by Proctor *et al*.^7^ Components included a five-day training on implementation science, mentoring, visiting implementation research sites, funding for pilot research and a conference. The success of the programme may have been related to seven key principles: continual updating of the curriculum, continuous contributions to advances in the field, a long training period of two years, involvement of multidisciplinary fields, multilevel processes, national leadership and networking opportunities. Lastly, the sixth study outlined how powerful a coproduced lived-experience research training programme with cofacilitation by lived-experience and conventional researchers was.^2^ It demonstrated to trainees the potential for lived-experience leadership and equality in research; however, systemic power asymmetries persisted between conventional and lived-experience researchers. This suggested that conventional mental health researchers require further training on the value of lived-experience research and the establishment of partnership principles to help navigate this.^2^

Overall, less emphasis has been given in past capacity building initiatives to what may be called conventional and lived-experience mental health research capacity building and career pathways.^3^ Lived experience mental health researchers bring their lived expertise into their work, either in the form of personal experience of mental ill-health or experience of caring for family or kinship members with mental ill-health and sometimes both experiences. With the increased involvement of community members and researchers with lived-experience in mental health research, it follows that training and capacity-building efforts may need some renewal.^12^ While collaborative networks for early career researchers in different fields have long existed,^13^ few detail investments made in mental health researcher and mental health lived-experience researcher infrastructure.^12^ Publications about lived-experience in mental health research and translation^14^ have grown, but there does not appear to be specific initiatives aimed at capacity building.^15^ Models providing skills development and training in lived-experience research do exist, but few are delivered at-scale and address how to embed coresearch models.^16^

In response, the Australian-based ALIVE National Centre for Mental Health Research Translation established a national, cross-disciplinary Next Generation Researcher Network (NGRN) with a tailored arm called the Lived-Experience Research Collective (referred to as the Collective) in 2022. Funded by the Australian National Health and Medical Research Council (2021–2026: GNT2002047), the Centre is in its fifth year and operates across 17 Australian universities and six founding service and peak policy organisations. The Centre is uniquely positioned in the implementation of approaches to address missing life years in priority populations (for example, First Nations communities and culturally diverse settings) due to unmet physical health needs^17^ through new models of care, systems redesign, improvement of experiences of care and the growth of future mental health research leaders. The NGRN and Collective, alongside the Co-Design Living Labs Network^18^ and the Implementation and Translation Network, support the operationalisation of six flagship research implementation projects (link: https://alivenetwork.com.au/our-research/our-projects/). The networks and research activities reflect a nexus model—a series of interconnected parts—that we propose may facilitate (1) meaningful integration of lived-experience perspectives and coresearch models, (2) collaboration across fields of research, policy and practice and (3) prioritisation of community-centric needs to implement research that addresses the priorities of people most impacted.

## The ALIVE National Centre capacity building strategy

The components of the Centre’s capacity building strategy are shown in online supplemental file 1. These are built on effective components identified in the preliminary literature review and follow a review of the common reasons shared by members for joining. Based on the frequencies of mentions, the main reasons for joining included ‘networking and collaboration, learning and improving skills, system reform and making meaningful change’. For the Collective, in addition to these shared areas, the common reasons for joining were ‘using lived-expertise to contribute to research/help others’. Network and Collective members also wanted to engage in ‘co-design and collaboration’ and to ‘focus on translating research to real people’. Thus, initiatives have been established since 2022 to respond to these reasons, building on effective approaches described in the literature.

**Figure 1:**
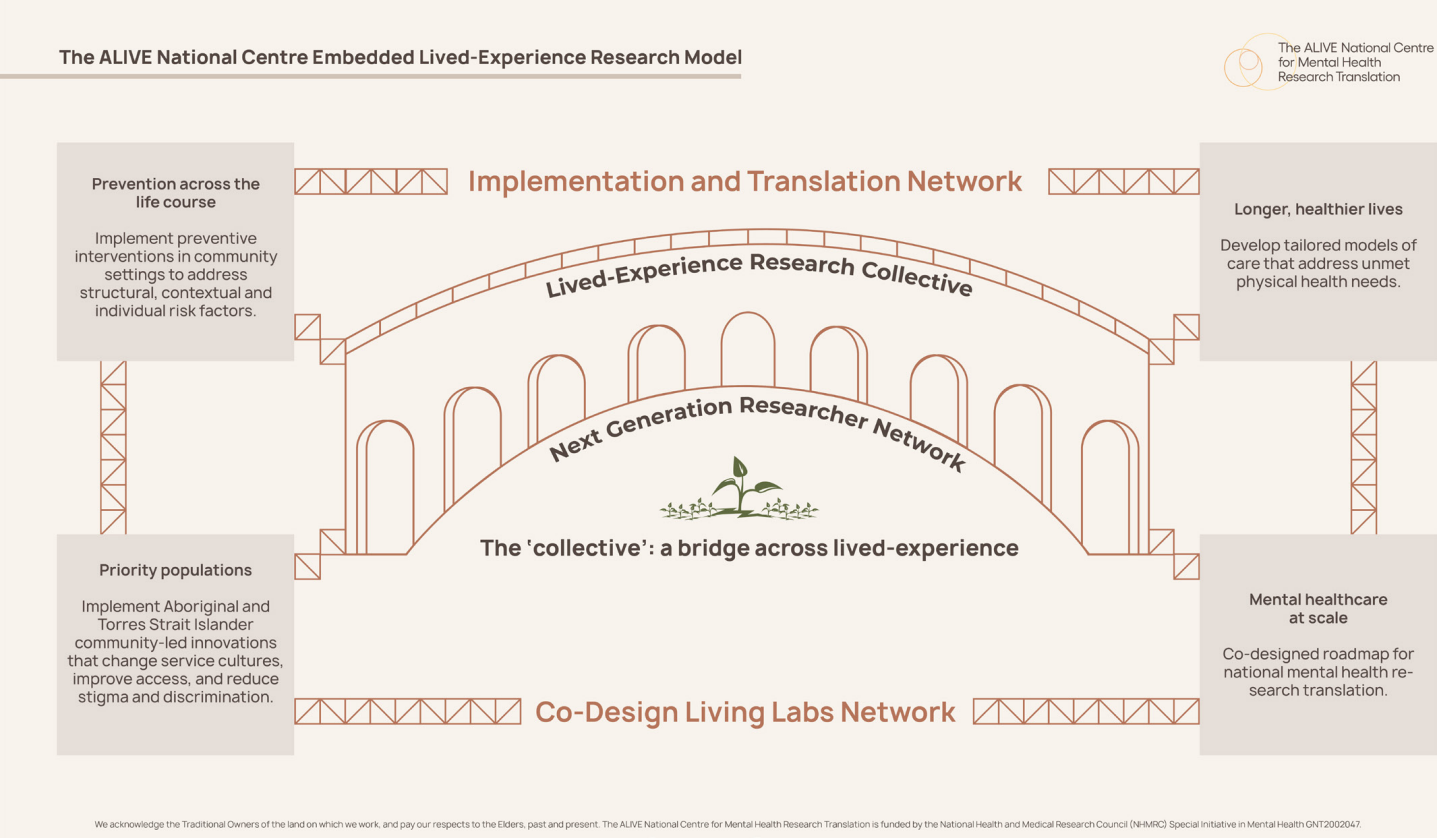
The ALIVE National Centre Embedded Lived Experience Research Model

At the governance and administration level, a Culturally Responsive Adaptive Governance Framework ensures collaboration across sectors, multisites and service settings and that the network is responsive to diversity of needs.^19^ This is supported by regular meetings between interdisciplinary coleads (with lived-experience researchers embedded) to develop network aims and goals. The Centre seeks to establish equity in Aboriginal and Torres Strait Islander research, also referred to as First Nations in health research, which forms a major programme of research due to disparities in the life years experienced by First Nations people.^17^ The central coordination hub reduces administrative burden for coleadership, and links sites and activities together; a method that has also been reported previously to enable successful implementation of multisite programmes.^1^ The coleadership model ensures the network is led by those for whom it was created, not only to have a voice for the broader membership but to build leadership skills, drive network activities and future research advocacy. The embedded lived-experience research model shown in figure 1 ensures that all lived-experience research collective coleads with direct personal experience and carer, family and kinship group experiences are embedded in the fabric of the Centre. This means that new coresearch models are implemented within research projects projects a service-research copartnership that has examined the implementation of cost-free, referral-free, community mental health service models in Australia with embedded peer workforces. The result was an Implementation Co-Evaluation Framework for future mental health research design and translation using lived-experience coresearch models (https://alivenetwork.com.au/wp-content/uploads/2024/09/Implementation-CoEvaluation-Framework-Design-Snapshot.pdf).

As part of the network infrastructure and strategy an annual seed funding scheme, tailored workshops and short courses, bursaries to attend courses in person run by the Centre, cocreated resources, a private online discussion forum, paid work opportunities and traineeships have been implemented. Future agendas will grow industry placements, with Implementation and Translation Network service members and expanded mentorship opportunities. Cross-collaboration between researchers with dual/multiple network memberships is a goal for the diverse research disciplines across disability, social work, psychology, rural mental health, policy, housing, child abuse, suicide, peer recovery, health economics, psychiatry and mad studies (a field of research that has its roots in the psychiatric and survivor movements and takes an activist and social justice lens).^20^

## Future directions

In the future, attention to who is not reached within the Next Generation Researcher Network and the Lived-Experience Research Collective will be important. Embedded lived-experience models that centre coresearch approaches will need to connect with First Nations capacity building efforts and explore the decolonisation of mental health research methodologies. Frameworks for cross-disciplinary approaches to complex problems will enable networked infrastructure for universities to work with service settings and to embed lived-experience in coresearch approaches in teaching and research. Opportunities exist for Next Generation Researcher Network members to participate in community exchanges within the Implementation and Translation network service organisation members. The goal is to improve practices in mental health and associated service settings, and experiences of care and enhance the future skills of mental health researchers. Consideration of how funding structures create barriers to interdisciplinarity is essential to address the siloing between social science, medical and health research funding schemes. Ultimately, a continuous, coordinated and centralised approach to strengthening the capacity of emerging mental health researchers will be instrumental in driving the necessary transformative change. A planned evaluation will establish the Network infrastructure success in preparing for mental health research of the future and achieving the best possible outcomes for people living with mental ill-health and carer, family and kinship groups.

## supplementary material

10.1136/bmjment-2025-301554 online supplemental file 1

## References

[1] Bartels SJ, Bruce ML, Unützer J (2013). Developing the next generation of researchers in emerging fields: case study of a multisite postdoctoral research training program. Acad Psychiatry.

[2] Bellingham B, Kemp H, Boydell K (2021). Towards epistemic justice doing: Examining the experiences and shifts in knowledge of lived experience researchers over the course of a mental health research training programme. Int J Ment Health Nurs.

[3] Evans-Lacko S, Hanlon C, Alem A (2019). Evaluation of capacity-building strategies for mental health system strengthening in low- and middle-income countries for service users and caregivers, policymakers and planners, and researchers. BJPsych Open.

[4] Hadidi NN, Lindquist R, Buckwalter K (2013). Lighting the fire with mentoring relationships. Nurse Educ.

[5] Kupfer DJ, Schatzberg AF, Dunn LO (2016). Career Development Institute with Enhanced Mentoring: A Revisit. Acad Psychiatry.

[6] Okewole H, Merritt C, Mangezi W (2020). Building Career Development Skills for Researchers: A Qualitative Study Across Four African Countries. Ann Glob Health.

[7] Proctor EK, Landsverk J, Baumann AA (2013). The implementation research institute: training mental health implementation researchers in the United States. Implement Sci.

[8] Renwick L, Keliat BA (2017). Implementing an innovative intervention to increase research capacity for enhancing early psychosis care in Indonesia. Psychiatric Ment Health Nurs.

[9] Rush SC, Wheeler J (2011). Enhancing junior faculty research productivity through multiinstitution collaboration: Participants’ impressions of the school psychology research collaboration conference. Can J Sch Psychol.

[10] Schneider M, van de Water T, Araya R (2016). Monitoring and evaluating capacity building activities in low and middle income countries: challenges and opportunities. Glob Ment Health (Camb).

[11] Sensoy Bahar O, Cavazos-Rehg P, Ssewamala FM (2021). Training LEADers to Accelerate Global Mental Health Disparities Research (LEAD) Program: A Research Training Program Protocol. Front Public Health.

[12] Thornicroft G, Cooper S, Bortel TV (2012). Capacity building in global mental health research. Harv Rev Psychiatry.

[13] Price E, Coffey B, Nethery A (2015). An early career academic network: what worked and what didn’t. J. Furth. High. Educ.

[14] Callard F, Rose D, Wykes T (2012). Close to the bench as well as at the bedside: involving service users in all phases of translational research. Health Expect.

[15] McEvoy PM, Horgan B, Eadon OL (2023). Development of a research capacity and culture tool for people with lived experience of mental health challenges. *Aust N Z J Psychiatry*.

[16] Loughhead M, Hodges E, McIntyre H (2024). Pathways for Strengthening Lived Experience Leadership for Transformative Systems Change: Reflections on Research and Collective Change Strategies. Health Expect.

[17] Angell B, Eades S, Jan S (2017). To Close the Gap we need to identify the best (and worst) buys in Indigenous health. Aust N Z J Public Health.

[18] Palmer VJ, Bibb J, Lewis M (2023). A co-design living labs philosophy of practice for end-to-end research design to translation with people with lived-experience of mental ill-health and carer/family and kinship groups. Front Public Health.

[19] Duke DLM, Prictor M, Ekinci E (2021). Culturally Adaptive Governance-Building a New Framework for Equity in Aboriginal and Torres Strait Islander Health Research: Theoretical Basis, Ethics, Attributes and Evaluation. Int J Environ Res Public Health.

[20] Beresford P (2020). ‘Mad’, Mad studies and advancing inclusive resistance. *Disability & Society*.

