# Supplementary material for: Capacity-building strategy for next-generation mental health research: embedding a national network infrastructure to grow mental health researcher capabilities and mental health lived-experience research leaders
Source: BMJ Ment Health. 2025 Mar 25;28(1):e301554. doi: 10.1136/bmjment-2025-301554 (PMC11938221; doi:10.1136/bmjment-2025-301554)
Supplement: online supplemental file 1 [file bmjment-28-1-s001.docx]

**Supplementary File 1. The component parts of the ALIVE National Capacity Building Strategy for future mental health research**

| **Component parts of the strategy** | **Mechanisms or ingredients that enact each component** | **Intended Outcomes** |
| --- | --- | --- |
| National Centre coordinating HUB for memberships, meetings and gatherings, bespoke activities, learning events and short course training for the Next Generation Researcher Network and the Lived-Experience Research Collective members. | -The HUB processes membership applications. A welcome pack is sent to new members with log in details to access a private peer online discussion forum. A central team member is responsible for coordination of the co-lead meetings, including facilitating open discussions.  -The HUB prepares communications about activities, events and training and disseminates this to members and across social media networks.  -The HUB tracks engagement with events and social media, and attendance at network meetings and gatherings and related activities. | *-Reduced administrative burden* for co-leads of the Network and Collective.  *-Upskilled mental health researchers* and lived-experience researchers from purposefully designed events and training.  *-Increased networking opportunities* and *collaboration* by fostering cross disciplinary dialogues and future collaborations of early and mid-career researchers. |
| Private Online Peer Discussion Forum on the Centre Digital Platform – A private members only space for peer research conversations and advancement of research questions and dialogue on flagships, or other topics of member interest. | -A moderated discussion forum is available to Next Generation Researcher Network and Lived-Experience Research Collective members on the ALIVE National Centre digital platform – each has an independent discussion forum and topics from quarterly virtual research meetings for Centre updates, questions raised at Lived-Experience Research Collective gatherings (where members meet virtually bimonthly) and network events can be discussed here. | -*Sense of peer community forming online*  Forum topics are discussed and engaged with consistently by member communities without prompting from research leads, co-director or network and Lived-Experience Research Collective co-leads. Sense of connections and collaborations form from online discussion space. |
| Responsive workshops, training (short courses) and activities across the Next Generation Researcher Network and the Lived-Experience Research Collective | i-LEARN Virtual Learning Studio Program - Integrated Lived-Experience Applied Research Nationally  -Annual Funding Galaxy Q&A (panel discussion event)  -Annual e-Networking event (opportunity for members to meet and network online) | -*Improved skill sets in mental health lived-experience research approaches and models* collaborations, grant success.  -*Shared knowledge* with each other and have a chance to ask experts in the field questions  *-Reduced power asymmetries* through dual events and activities between Next Generation Researcher Network, Lived-Experience Research Collective as well as the broader Centre networks and groups. |
| Centre’s Adaptive Research Governance Framework supports effective implementation of research to meet Centre outcomes, program objectives, monitoring and tracking and of establishing wider translational pathways.   This includes the embedded model of lived-experience research. | -The governance framework includes an Independent Advisory Board (IAB), Intersectoral Policy and Practice Committee (IPPC), International Scientific Advisory Committee (ISAC) and Executive Research Leadership Committee.    -There are co-chairs with lived-experience across all Boards and Committees.   -Co-leads of the Lived-Experience Research Collective are embedded across all networks.  -Co-leads facilitate gathering meetings of the Lived-Experience Research Collective held every second month and participate in the quarterly research forums of the Centre.  -Co-leads are included within research grant applications into the future.  -Members of the Lived-Experience Research Collective have opportunities for paid research roles to grow embedded co-research models and to increase track records and collaborative ways of working. | *-Strengthened relationships between research, policy and practice* through intersectoral relationships and combined governance meetings.  - The adaptive framework promotes *equity in Aboriginal and Torres Strait Islander health research.*  *-Embedded lived experience within all levels of the Centre governance and its operationalisation.*    -*Increased interconnection* across the Centre’s network and research activities with opportunities provided for regular co-reflection on the embedded model.  *-Growth of leadership skills* and capabilities of lived-experience researchers with greater knowledge of research activities and processes.  *-Development of track records of mental health lived-experience researchers* through inclusion within grants, contracts, tenders and other research events and activities. |
| Establishment of internal funding schemes focussed towards early to mid-career researchers across mental health researchers and researchers with lived-experience | -Annual seed funding scheme for the first wave of National Health and Medical Research Council funding. Built in options for broad teams, lived-experience led research, Aboriginal and Torres Strait Islander led research and non-traditional research projects.    - Rolling PhD funding scheme (Launch Pad)  - Annual mid-career funding scheme (Professional Advancement and Career Expansion awards)  -In/formal mentoring opportunities and pathways  -Lived-Experience Research Collective representatives join generation researcher co-lead network meetings (alongside community members who bring lived-experience to research design and translation from the Co-Design Living Labs Network)  -Conference bursaries to support researchers to gain knowledge and attend conferences | *-Strengthen lived-experience researcher opportunities to lead projects* and create career pathways and tailored funding opportunities.  -Support PhD students develop their careers and provide financial support for activities involving people with lived-experience.  -Assist mid-career researchers in expanding their skill-sets, networks and career.  -*Development of career pathways* for lived-experience researchers that accommodate for non-traditional research training skills and entry points into research careers. |
| Co-leadership models have been implemented to grow lived-experience research leadership and activities. | -The NGRN has 12 co-leads (including 2 investigator co-leads and 2 Lived-Experience Research Collective co-leads). The Lived-Experience Research Collective has 12 co-leads (including 5 carer/family/kinship co-leads.)    -Co-lead meetings are facilitated by the HUB and National Centre funded research fellows.    -Co-leads increasingly facilitate members to take ownership and drive network activities and host events and presentations such as the virtual translation café series. | -Embedded co-leads across the networks and research programs leads to *increased lived-experience researcher engagement* in leadership and research governance.   -Evidence that co-leads have fostered member-based activities locally and nationally without prompts from the HUB and or research leaders in the Centre.  -Co-leads across capacity building networks have *increased visibility* across the sector leading to increased collaborative opportunities. |
| Community and industry engagement and skills development | -Peer Exchange Program  (mentoring for lived-experience researchers)  -Community Exchange Program (industry placements)   -Engagement with Implementation and Translation Network | -*Collaboration between university and industry,* improving research translation efforts.  -Early and mid-career researchers to be exposed to *multiple career pathways* not limited to academia. |
| Cross Network Collaborations within the National Centre for Mental Health Research Translation | -Dual memberships of NGRN and Lived-Experience Research Collective.  -Lived-Experience Research Collective representatives present at NGRN meetings (and across other network and research program meetings)  -National Centre Quarterly Forum (virtual centre update)  -National Centre Annual Symposium  (hybrid overview of year activities and future plans)  -Ready, Set, Translate – The ALIVE Mental Health Research Translation Virtual Café Series  (monthly virtual presentations by experts in the field)  -National Centre Zine: *“Lost in Translation”* and other creative communication outputs (quarterly centre magazine)  -Annual World Mental Health Day event (topic changes annually) | -*Improved capacity* as an implementation and translation centre.  -Evidence of *data sharing* for collaborative research  -Collaborative grant and publication submissions and successes.   -Continue successful engagement strategies and modify as needs indicate/demand. |
| Co-created resources developed and made available for long-term use | -“Co-Created Guide for Researchers Navigating the Mental Health Research Funding Galaxy” which is updated yearly following the funding panel Q&A: <https://go.unimelb.edu.au/qbk8>.  -“Co-Creating Knowledge: A Guide to the Mental Health Lived-Experience Research Landscape” (in formation) | -Enables activities to have a *lasting impact* and be used as tools by members throughout their careers. |
| Development of A National Strategy for Lived-Experience in Mental Health Research | -The Long Conversation Project- compiling the “who, what, where and how” of lived-experience researchers nationally (survey and creative outputs)  -Focused narrative review and synthesis of current processes, practices and principles, development of a typology how lived-experience in mental health research is described, reported and where available evaluated.  -Co-Design of the typology further from lived-experience researchers and carer, family and kinship group researchers, interviews and focus groups with organisations, policy makers and funders about the strategy development and its implementation.  -Draft strategy for open consultation. | -*Defined roles* across research institutions and other sectors where research activities are underway.  -Identification of organisational conditions within which people are working and the needs *for trauma-violence informed research collaborative practices.*  -Identification of the *capability’s development needed* across lived-experience research roles and approaches.  -Mapping of the processes, principles and practices of lived-experience in mental health research.  -Clear identification of *career pathway development* needs for implementation across research sectors. |
| Evaluation of impact and meeting the funding objective to grow the next generation of mental health research leaders and lived-experience research development. | -Embedded impact evaluation of Centre’s progression in a co-designed roadmap based on priorities of people with lived-experience and carer, family and kinship group members.   -A social return on investment evaluation of the Centre’s impact in terms of its internal initiatives for capacity building. | -That capabilities are developed to *support major priority area* of the Phase 1 Consensus Statement/; lived-experience in all aspects of research (<https://alivenetwork.com.au/phase-1-consensus-statement-the-short-horizon-2023-implementation-actions/>).   -*That the capacity building strategy delivers outcomes* for individual researchers for fellowships and grants, increased implementation and translation pathways, and independent research program development for Centre sustainability into the future. |
